# Oval Cell Response Is Attenuated by Depletion of Liver Resident Macrophages in the 2-AAF/Partial Hepatectomy Rat

**DOI:** 10.1371/journal.pone.0035180

**Published:** 2012-04-13

**Authors:** Shuai Xiang, Han-Hua Dong, Hui-Fang Liang, Song-Qing He, Wei Zhang, Chang-Hai Li, Bi-Xiang Zhang, Bin-Hao Zhang, Kai Jing, Stephen Tomlinson, Nico van Rooijen, Li Jiang, Katherine Cianflone, Xiao-Ping Chen

**Affiliations:** 1 Hepatic Surgery Centre, Huazhong University of Science and Technology, Tongji Hospital, Wuhan, China; 2 Department of Hepatobiliary Surgery, Guilin Medical University, Affiliated Hospital, Guilin, China; 3 Department of Microbiology and Immunology, Darby Children's Research Institute, Medical University of South Carolina, Charleston, South Carolina, United States of America; 4 Department of Molecular Cell Biology, Vrije Universiteit Medical Center, Amsterdam, The Netherlands; 5 Centre de Recherche Institut Universitaire de Cardiologie et de Pneumologie de Quebec, Université Laval, Quebec, Canada; Agency for Science, Technology and Research - Singapore Immunology Network, Singapore

## Abstract

**Background/Aims:**

Macrophages are known to play an important role in hepatocyte mediated liver regeneration by secreting inflammatory mediators. However, there is little information available on the role of resident macrophages in oval cell mediated liver regeneration. In the present study we aimed to investigate the role of macrophages in oval cell expansion induced by 2-acetylaminofluorene/partial hepatectomy (2-AAF/PH) in rats.

**Methodology/Principal Findings:**

We depleted macrophages in the liver of 2-AAF/PH treated rats by injecting liposome encapsulated clodronate 48 hours before PH. Regeneration of remnant liver mass, as well as proliferation and differentiation of oval cells were measured. We found that macrophage-depleted rats suffered higher mortality and liver transaminase levels. We also showed that depletion of macrophages yielded a significant decrease of EPCAM and PCK positive oval cells in immunohistochemical stained liver sections 9 days after PH. Meanwhile, oval cell differentiation was also attenuated as a result of macrophage depletion, as large foci of small basophilic hepatocytes were observed by day 9 following hepatectomy in control rats whereas they were almost absent in macrophage depleted rats. Accordingly, real-time polymerase chain reaction analysis showed lower expression of albumin mRNA in macrophage depleted livers. Then we assessed whether macrophage depletion may affect hepatic production of stimulating cytokines for liver regeneration. We showed that macrophage-depletion significantly inhibited hepatic expression of tumor necrosis factor-*α* and interleukin-6, along with a lack of signal transducer and activator of transcription 3 phosphorylation during the early period following hepatectomy.

**Conclusions:**

These data indicate that macrophages play an important role in oval cell mediated liver regeneration in the 2-AAF/PH model.

## Introduction

Under conditions where proliferation of mature hepatocytes is inhibited, liver progenitor cells, also known as oval cells (OCs), expand and differentiate into mature hepatocytes and biliary epithelial cells in order to regenerate liver mass following hepatic damage. Liver progenitor cells are generally acknowledged to be involved in many human liver diseases and experimental animal models [1], [2], [3], [4]. Stem cell therapy has promise as a new approach for treating life-threatening liver disease. On the other hand, there is a potential downside to the use of liver progenitor cells because of their possible malignant transformation, which is well documented [5], [6], [7]. Thus, attention has recently focused on elucidating the responses and mechanisms involved in the activation and expansion of liver progenitor cells.

The microenvironment is highly regulated within an organ and plays an important role in maintaining stem cell division under signaling modulation [8]. The microenvironment comprises the extracellular matrix, epithelial and non-epithelial resident liver cells, and recruited inflammatory cells, as well as a variety of growth-modulating molecules. Innate immune cells and inflammatory processes have been reported to play an important role in OC dependent liver regeneration [9], [10], [11], [12].

Resident macrophages constitute a major non-parenchymal cell population of the liver. Liver resident macrophages or Kupffer cells (KCs) are in close contact with the sinusoidal endothelium and may reach into the space of Disse and near hepatocytes. Macrophages are one of the primary sources of inflammatory mediators in the liver, including the cytokines tumor necrosis factor-alpha (TNF-*α*) and interleukin-6 (IL-6). Several studies employing selective KC depletion in rodents have explored the role of KCs in hepatocyte proliferation and liver regeneration following partial hepatectomy [13], [14], [15], [16]. However, there is little information available on the role of macrophages in OC expansion. In some OC expansion models, such as those involving a choline-deficient ethionine-supplemented diet, the administration of 2-acetylaminofluorene (2-AAF) plus partial hepatectomy (PH), and CCl_4_/2-AAF injury, an expansion in macrophage numbers were observed [9], [17], [18]. Usually, macrophages were present in greater numbers in the periportal area compared to near the central vein [19]. Thus, there is a good reason to hypothesize that macrophages may influence the development of OCs during liver regeneration.

To test this hypothesis, we investigated the specific contribution of macrophages on OC mediated liver regeneration. The role of KCs in the regulation of OCs has been previously investigated [20]. In that study, Gadolinium chloride (GdCl_3_) was used as the macrophage suppressing agent together with bile duct ligation to induce injury. However, the bile ductular reaction from bile duct ligation is considered to be a different cell type from OC [21], [22], [23], [24]. In addition, GdCl_3_ interferes with macrophage function and does not result in macrophage ablation [25], [26]. In this study, we employed the 2-AAF/PH model in which liver regeneration is mediated by OC expansion and differentiation into mature hepatocytes. This method was combined with macrophage depletion using liposome encapsulated clodronate (Lip-Clod) which causes specific depletion of resident liver macrophages [27], [28]. We report here that liver resident macrophages play an important role in OC proliferation and differentiation.

## Results

### Depletion of resident macrophages

**Figure 1:**
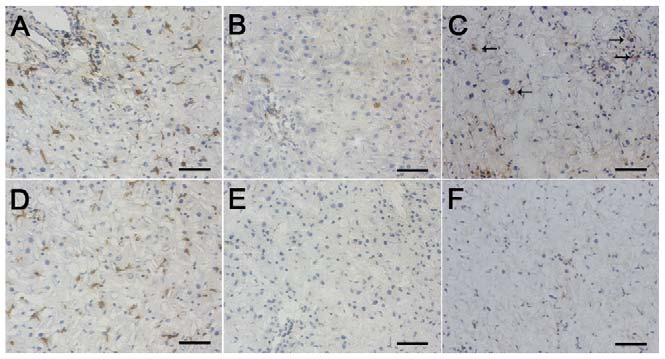
Kupffer cells were depleted by treatment with Lip-Clod. ED1 immunostaining of rat liver sections (A, B and C, ×200). ED2 immunostaining of rat liver sections (D, E and F, ×200). ED1 (A) and ED2 (D) positive cells could be easily identified in sinusoidal areas in control rats. Only a few ED1 positive cells could be observed 2 days after Lip-Clod administration (B). Some small ED1 positive cells appeared 6 days after PH (C). Arrows point to positive cells. Treatment with Lip-Clod resulted in a nearly complete elimination of ED2 positive Kupffer cells 2 days after Lip-Clod administration (E). Few ED2 positive cells could be identified 9 days after PH (F). Bar = 50 *µ*m.

All rats received 2-AAF/PH treatment as oval cell model, and were then randomly divided in two cohorts, control group or macrophage-depleted group. Rat liver resident macrophages were depleted by the so-called “suicide” technique, i.v. injection of Lip-Clod via tail vein two days before PH [27], [28]. To confirm that the macrophages had been depleted, frozen liver sections collected from each time point were stained with the monoclonal antibodies for ED1 and ED2, and positive cells were counted. Quantified data of the trend of ED1 and ED2 positive cells were shown in Table 1. Two days after clodronate treatment, ED2 positive macrophages were virtually absent (Figure 1E), and only a few ED1 positive cells were observed (Figure 1B). Some newly positive ED1 cells with a small size could be observed 6 days after PH. ED2 positive cells remained almost completely absent until day 9 after PH (Figure 1F), at which time point only 0.9±0.5 ED2 positive cells per 0.4×0.32 mm^2^ field could be observed in clodronate-treated rats versus 21.7±1.3 in the control rats (*P*<0.01), which is similar to our previous findings [29]. The newly reappeared ED1 positive cells 6 days after PH represent immature macrophage populations. Therefore, one dose of Lip-Clod can efficiently deplete resident macrophages for up to 9 days, and ED1 positive cells during the early phase after PH.

**Table 1:** Quantification of ED1 and ED2 positive cells in the liver.

		0 d post PH	4 d post PH	6 d post PH	9 d post PH
ED1	Control	17.4±3.3	34.0±2.5	26.0±2.3	24.2±4.2
	Lip-Clod	3.3±1.4**	4.2±1.4**	13.3±2.8**	18.1±3.0*
ED2	Control	17.3±0.9	27.5±3.0	25.8±1.7	21.7±1.3
	Lip-Clod	0.8±0.5**	1.0±0.5**	0.9±0.2**	0.9±0.5**

Data represent mean ± SD, n = 4–5.

* *P*<0.05;

** *P*<0.01 compared to respective controls for each time point.

### Mortality and liver damage is increased, and remnant liver regeneration is attenuated in macrophage depleted 2-AAF/PH rats

**Figure 2:**
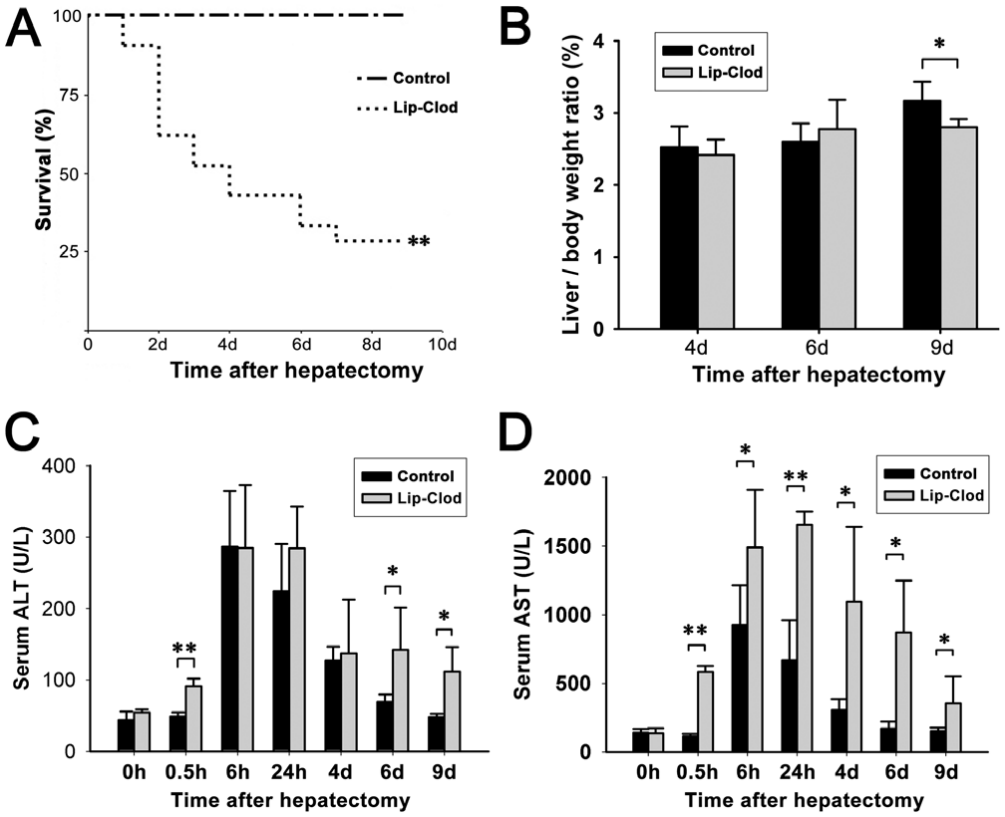
Effect of KC depletion on liver damage and regeneration and rat survival after PH. Accumulated survival rate (A). liver weight/body weight ratio (B). Blood serum levels of ALT and AST (C and D). (Data represent mean ± SD, n = 4–5. * *P*<0.05; ** *P*<0.01).

2-AAF/PH rats (n = 4–5) were killed in each group at 0, 0.5, 6, 24 hours and 4, 6, 9 days after PH. Body weight and liver weight were recorded. All 2-AAF/PH control rats treated with saline survived for the time period studied. However, in 2-AAF/PH rats that were depleted of macrophages, a significant reduction in body weight gain post PH was observed when compared to control rats (data not shown), along with an unexpectedly high mortality rate (Figure 2A). Only 30% of rats in the macrophage depleted group survived beyond 9 days. To eliminate the influence of possible differential body weight change, liver weight/body weight ratio was used to assess remnant liver restoration. This ratio in macrophage-depleted rats was significantly reduced when compared with that in control rats treated with saline at 9 days after PH (Figure 2B). We next assessed liver damage by measuring serum levels of alanine transaminase (ALT) and aspartate aminotransferase (AST). We showed that ALT and AST levels increased 6 hours after PH and decreased at the following time points in control rats. Compared to control rats, macrophage-depleted rats had a significantly higher peak level of AST, as well as earlier and more prolonged elevation of ALT and AST (Figure 2, C and D).

### Oval cell amplification is attenuated in macrophage depleted 2-AAF/PH rats

**Figure 3:**
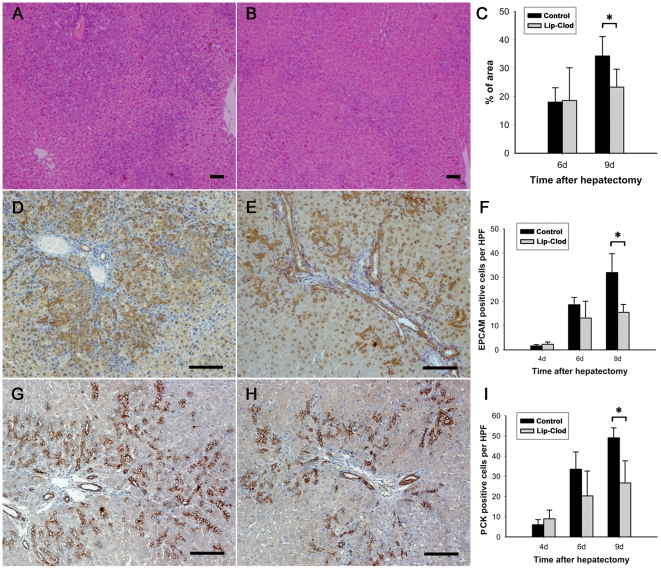
Identification of oval cell expansion in liver sections of 2-AAF/PH rats. A and B: HE staining showing periportal ductular reactions from control (A) and KC depleted rats at day 9 after PH (B) (×40). Comparison of percentage of ductular reaction areas in different groups of animals (C). Data expressed as percentage of ductular reaction area to total area. Immunostaining with antibodies against EPCAM (D and E) and PCK (G and H) in paraffine embedded liver sections from control (D and G) or KC depleted (E and H) rats 9 days after PH (×100). Bar = 100 µm. F and I: Comparison of the number of EPCAM (F) or PCK (I) positive oval cell in different groups of animals. (Data represent mean ± SD, n = 4–5. * indicates significant difference when compared to controls group, *P*<0.05).

In control rats, OCs were most abundant at day 9 after PH. However, in macrophage depleted rats there was a significant decrease in OC response. Periportal ductular reaction areas were measured from hematoxylin-eosin (HE) stained sections. Areas were calculated by tracing the periportal basophilic area borders. We measured percentage of periportal basophilic area by using the formula: (basophilic area ÷ total area)×100%. The periportal basophilic foci represented OC clusters. We founded that in macrophage depleted rats the ductular reaction only covered 23.31% of total area compared to 34.29% in control rats 9 days after PH (*P* = 0.03; Figure 3, A–C). Then, we performed immunohistochemistry for OC marker epithelial cell adhesion molecule (EPCAM) and pan cytokeratin (PCK), and analyzed positive cells. Quantification of cells positive for EPCAM (Figure 3, D–F) and PCK (Figure 3, G–I) demonstrated that less OCs were present in the macrophage depleted rats on day 9 compared to control rats. To rule out any potential direct toxic effect of Lip-Clod on liver cells, we cultured two liver cell lines L02 and LE/6 with Lip-Clod or PBS in vitro. Lip-Clod had no effect on the viability of either cell line as determined by 3-(4,5-dimethyl-2-thiazolyl)-2,5-diphenyl tetrazolium bromide (MTT) assay. (data not shown)

### Attenuated OC amplification is not due to attenuated proliferation or increased apoptosis

**Figure 4:**
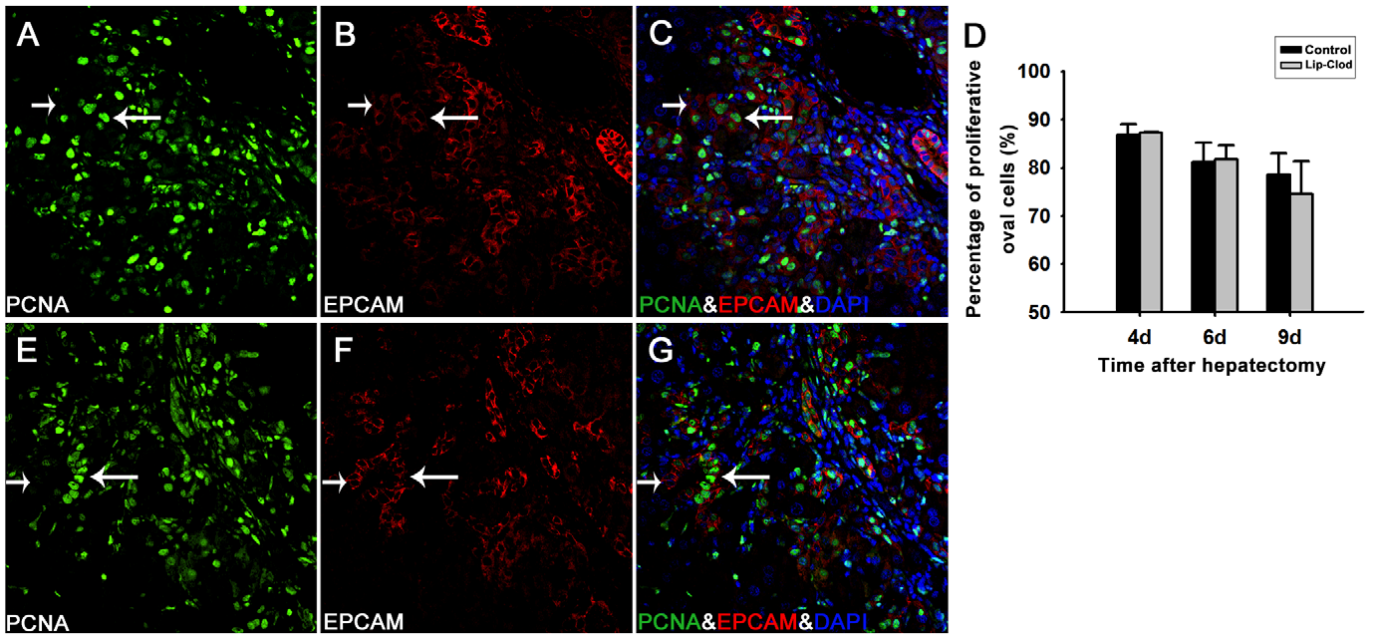
Oval cell proliferative activity as assessed by percentage of EPCAM positive cells labeled with PCNA. Double immunofluorescent staining for PCNA (green) and EPCAM (red) in the portal area was performed in liver sections from KC non-depleted (A–C) and depleted (E–G) 2-AAF/PH treated rats 6 days after PH (×400). PCNA positive (long arrow) and negative (short arrow) oval cells can be easily distinguished and counted. D: Percentage of proliferating oval cells was not significantly different between the two groups on days 4, 6, and 9 after PH. *P*>0.05. (Data represent mean ± SD, n = 3–4).

**Figure 5:**
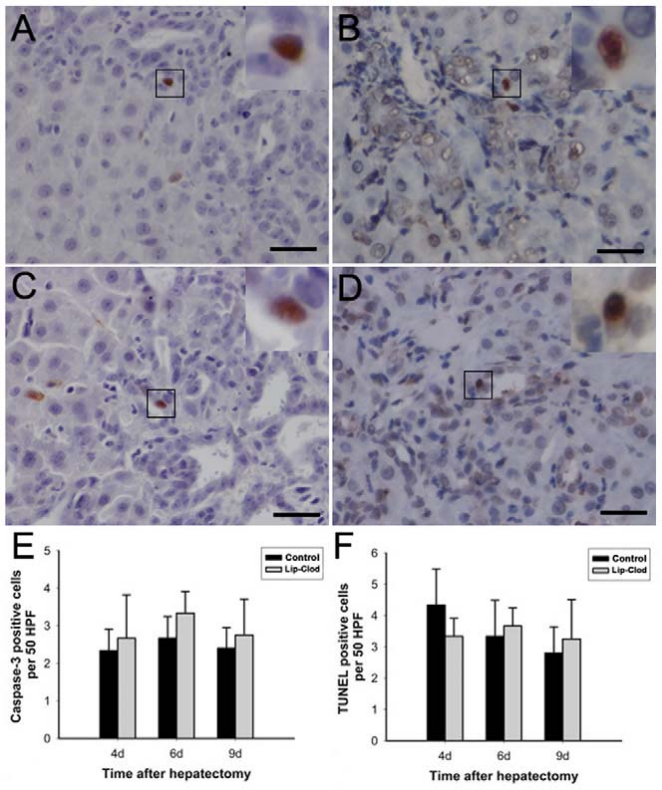
Oval cell apoptosis as assessed by caspase-3 immunohistochemistry and TUNEL analysis. (A and B) show apoptotic oval cells from a control rat 9 days after PH. (C and D) show apoptotic oval cells from a KC-depleted rat 9 days post PH. Comparison of the number of caspase-3 or TUNEL positive cells in different groups of animals (E, F). The top right insets in all images show high magnification of bracketed areas. Bar = 25 µm. There was no significant difference between the two groups on days 4, 6, and 9 after PH. *P*>0.05. (Data represent mean ± SD, n = 4–5).

To facilitate quantification of OC proliferative activity, double immunofluorescent staining for PCNA and EPCAM was performed. The percentage of proliferative OCs was similar in macrophage depleted and control non-depleted rats (Figure 4). Furthermore, we did not observe any difference in the number of apoptotic periportal OCs between the two groups as determined by both Terminal Deoxynucleotidyl Transferase-Mediated dUTP Nick-End-Labeling (TUNEL) analysis and caspase-3 immunohistochemistry (Figure 5).

### Macrophage depletion attenuates OC differentiation in 2-AAF/PH animals

**Figure 6:**
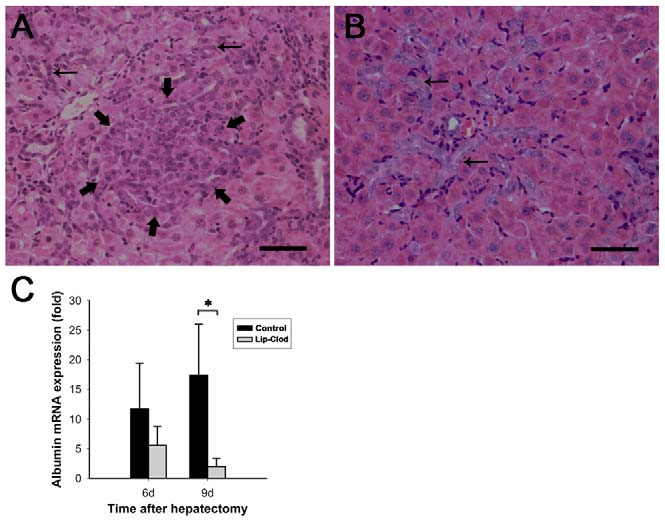
Kupffer cell depletion affects oval cell differentiation on day 9 after PH. HE staining (A, B). Foci of small hepatocyte-like cells (short arrows) could be easily identified in non-KC depleted rats 9 days after PH (A ×200). Oval cells showed typical oval cell morphology without obvious change in KC depleted rats 9 days after PH (B ×200). Bar = 50 µm. Expression of albumin transcripts was evaluated by real-time quantitative RT-PCR (C). KC depletion resulted in decreased expression of albumin on day 9 after PH. Arrows point to oval cells. (Data represent mean ± SD, n = 4–5. *indicates significant difference when compared to control group, *P*<0.05).

The appearance of small hepatocyte-like cells is a feature of OC differentiation to hepatocytic-lineage in 2-AAF/PH rats [30], [31], [32]. In comparison to the classical morphology of OCs, these cells are bigger with a rounder nucleus and a lower nuclear-to-cytoplasm ratio. In this study, large populations of small hepatocyte-like cells were observed migrating in clusters from the ductular reaction area into the parenchyma by day 9 in the control group (Figure 6A). By contrast, fewer of these cells were observed in the macrophage depleted group over the measured 9 day period after PH (Figure 6B). This finding indicates that depletion of macrophage in the 2-AAF/PH model affects OC differentiation.

To further investigate differential OC differentiation, expression of albumin mRNA was subsequently analyzed via Real-time polymerase chain reaction (PCR) in macrophage depleted and non-depleted rat livers. Albumin mRNA expression in non-depleted rat livers was significantly higher by day 9 after PH (Figure 6 C).

### Macrophage depletion reduces hepatic TNF-*α* and IL-6 expression and inhibits signal transducer and activator of transcription 3 (STAT3) activation

**Figure 7:**
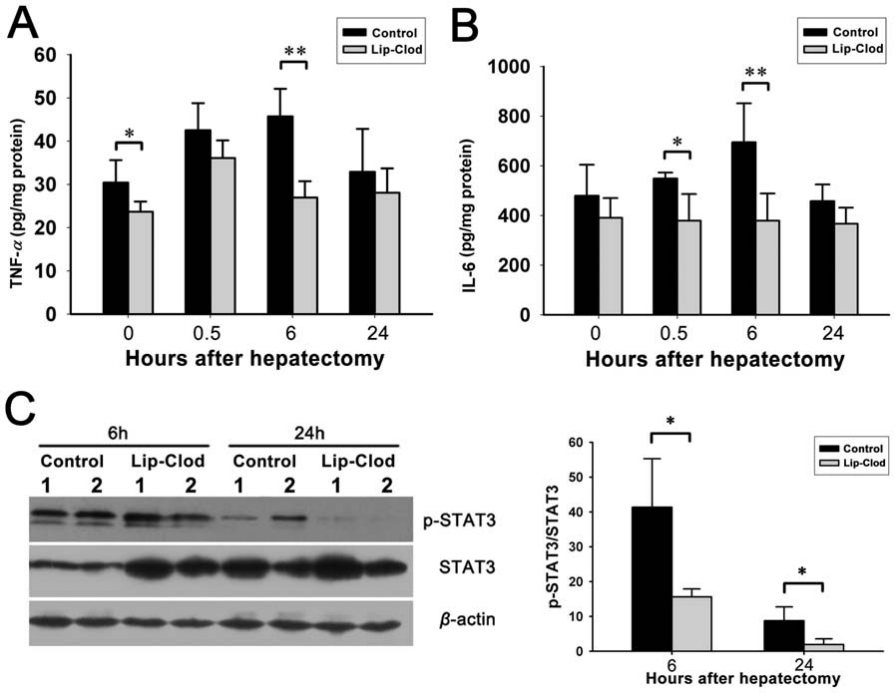
Effect of KC depletion on the expression of TNF-*α*, IL-6, and activation of STAT-3 protein in liver tissue after PH. ELISA was performed on supernatants from liver tissue homogenates (A, B). Western blot analysis of total and phosphorylated STAT3, including *β*-actin as a loading control (C). KC depletion reduced hepatic levels of TNF-*α* and IL-6, and reduced STAT3 activation. (Data represent mean ± SD, n = 4–5. * indicates significant difference when compared to control group, *P*<0.05; ** *P*<0.01).

Macrophages play an important role in stimulating OCs by releasing several important cytokines such as TNF-*α* and IL-6 [33]. Then we assessed whether there were differences in the levels of these cytokines in livers. Liver tissues were homogenized in extraction buffer, and levels of TNF-*α* and IL-6 in the supernatants were detected using enzyme-linked immunosorbent assay (ELISA). During the early OC activation period, there was an increase in hepatic TNF-*α* and IL-6 levels, with a peak at 6 hours after PH in control rats. However, both cytokines were significantly reduced in macrophage depleted rats, and there was no increase in IL-6 after PH in this group (Figure 7, A and B). IL-6 plays an important role in the induction of the JAK-STAT3 pathway that contributes to hepatoprotective and hepatomitogenic effects in liver regeneration [34], [35]. We, therefore, also determined whether macrophage depletion affects STAT3 activation. Western blotting was performed to detect liver expression of STAT3, phosphor-STAT3 and *β*-actin. We found that at 6 and 24 hours after PH, phosphorylation of STAT3 was significantly reduced in macrophage depleted rats (Figure 7C).

## Discussion

Macrophages can produce and secrete many kinds of cytokines, which play important roles in liver injury and regeneration. Selective depletion of macrophage is a useful and widely accepted approach to investigate their function in vivo. However, data concerning the role of KCs in liver regeneration remains controversial. Some findings suggest that KCs play a stimulatory role in liver regeneration [13], [14], while inhibitory roles have been described by other investigators [15], [16]. This may be due to complex and multi-functional roles of KCs in liver regeneration, as KCs are a major source of both pro-proliferative and anti-proliferative mediators in the liver. Different reagents have been employed for the purpose of depleting macrophages, and these varying techniques may affect macrophage function/depletion in many ways, as well as the function/depletion of other cells. One methodology frequently used in the past to deplete macrophage involves the use of GdCl_3_. However, this may not be a suitable technique since GdCl_3_ is highly toxic and is retained in hepatocytes. Moreover, whether GdCl_3_ eliminates macrophage by depletion or alteration of their function and phenotype is controversial [36], [37]. Thus, the use of GdCl_3_ to induce macrophage depletion leads to substantial difficulties in data interpretation. A more reliable reagent for macrophage depletion is Lip-Clod, since (1) liposomes are immunologically inert, (2) released clodronate does not easily enter cells, (3) free clodronate exhibits a very short half-life in both the circulation and body fluids, and (4) Lip-Clod is non-toxic and selectively eliminates macrophage without macrophage activation [28]. We, therefore, chose this approach to achieve macrophage depletion and demonstrated that Lip-Clod treatment was highly effective, with a nearly complete elimination of ED2 positive cells in the liver during the period observed, and ED1 positive cells during the early period after PH.

We measured serum ALT and AST as indicator for liver function and liver damage. Both hepatic enzymes were comparable between the two groups at the time of PH (0 hour). We excluded any direct effect of Lip-Clod on the viability of liver cells in vivo or in vitro [38]. This indicates that depletion of macrophages does not induce liver injury following administration of a unique dose of 2-AAF. Exposure to PH resulted in increases in ALT and AST level in both groups. However, increased leakage of ALT and AST was recognized immediately after PH at 0.5 hour in macrophage-depleted rats, which is earlier than that in control rats. Moreover, a higher peak value of AST level and a prolonged elevation of both transaminase levels were identified in the Lip-Clod group. These finding may be explained by the fact that resident macrophages exert a protection role in liver injury [37]. In addition, restoration of remnant liver mass in 2-AAF/PH rats with macrophage depletion, was slower 9 days after PH. This result is concordant to the delayed recovery of serum transaminase levels. Taken together, these data indicate that macrophage depleted rats were less able to compensate for the damage in the 2-AAF/PH liver injury model, in which liver regeneration is mainly mediated by OCs. Accordingly, 2-AAF/PH rats had a significantly higher mortality rate in the absence of macrophages.

Oval cell proliferation in the 2-AAF/PH model was measured using several parameters, including evaluation of the periportal basophilic area of newly formed ductular reaction and the number of OCs identified by two commonly accepted markers, EPCAM and PCK. A significant attenuation of OC response was observed at 9 days after PH in macrophage depleted rats. These indicate that OC expansion in response to 2-AAF/PH injury is impaired by macrophage depletion, suggesting a possible role of resident macrophages in OC proliferation. Unexpectedly, we did not find any significant difference in OC proliferation ratio and apoptosis between the two groups of rats (Figure 4 and 5). These results suggest that absence of macrophages may not affect the kinetics of OC development after initiation of cell cycle. Interestingly, we noticed that there was a trend of decreasing OC number (although not significantly different) until 9 days after PH in macrophage depleted rats, suggesting a mild difference of OC number between the two groups at an early time period. It is well known that oval cells exist in an extremely small number in the quiescent liver. In liver regeneration, OC need to be “primed” in the acute phase to enter the cell cycle before responding to the stimulatory effects of growth factors [39], and then undergo significant rounds of duplication during liver regeneration [4]. Thus, we propose that removal of the macrophage influence before PH primarily affects OCs at a very early time point, and the mild difference in OC number at the early phase may be amplified manyfold in the later time point after significant rounds of proliferation.

Macrophages exert a stimulatory effect mainly by releasing TNF-*α* and IL-6, which are considered to be the initiators of progression of liver regeneration [40], [41]. It has been documented that liver mRNA levels of TNF-*α* and IL-6 increase in the early period immediately after PH [24]. Under circumstances where both of these cytokines are inhibited by dexamethasone, OC induction and expansion in response to 2-AAF/PH is severely diminished [24]. Similarly, ethionine-supplemented diet induced OC proliferation is substantially impaired in TNFR1 deficient mice, and also in IL-6 knockout mice [42]. We then focused on the effect of macrophage-depletion on cytokine production within the acute phase after PH. In this study, we found that liver level of TNF-*α* was significantly reduced during the early phase after PH in macrophage depleted rats. This was accompanied by a strong inhibition of IL-6 elevation in the acute phase. We showed that IL-6 increased approximately 45% at 6 hour after PH in control rats compared to the basal level at 0 hour, but this increase was completely inhibited by macrophage depletion. STAT3 phosphorylation, mainly activated by IL-6, also was significantly down regulated in macrophage depleted rats. Thus, the reduced production of the components of regeneration stimulating pathway TNF–TNFR1–NF*κ*B–IL-6–STAT3 in macrophage depleted rats may be a potential mechanism explaining the decreased OC expansion. The effect of macrophage deletion on the other pathways (e.g. JNK, ERK and WNT pathway) [43], [44] which is important to oval cell proliferation needs further studies. Oval cell mediated liver regeneration is a systematic program, and other liver nonparenchymal cells also contribute to OC expansion. A recent report has revealed that CD11b^+^ invading macrophages play a major role in the OC response to chronic liver injury in ethionine-supplemented diet mice [45]. Indeed, Lip-Clod has no effect on depleting CD11b^+^ macrophages, which also possess potent cytokine-producing capacity [46]. This may be one of the reasons for the fact that macrophage depletion did not completely abolish OC response in 2-AAF/PH rats in this study.

Most of the OCs did not show any significant morphological alterations and were at a similar stage of differentiation during the early expansion between 2 days and 9 days after PH in this model [47]. Accordingly, we observed that small hepatocyte-like cells were present between ductular reactions and liver parenchyma in the control group at 9 days after PH. By contrast, OC maturation was impaired, since no prominent formation of small hepatocyte-like cells was found in the macrophage-depleted rats for up to at least 9 days after PH. Small hepatocyte-like cells have a considerably smaller nucleus than mature hepatocytes, and have a rounder nucleus than primary OCs. Small hepatocyte-like cells also form small basophilic foci at high cell density. It has been demonstrated that these new hepatocytes are a differentiating progeny of OCs [30], [31], [32]. Also the formation of small foci of basophilic hepatocytes is a very critical step in the differentiation of OCs [47]. Therefore, our findings suggest that depletion of macrophage inhibits or delays OC differentiation in a 2-AAF/PH model. Administration of 2-AAF can significantly reduce albumin expression in pre-existing mature hepatocytes [48]. Small basophilic hepatocytes, as a transition cell type from OCs to mature hepatocytes, show sharply increased expression of albumin [32], [48]. In this study, livers in control rats with apparent formation of basophilic foci showed significantly higher expression of albumin than in macrophage-depleted rats at 9 days after PH. These further indicate that OC differentiation in 2-AAF/PH rats is negatively affected by macrophage depletion.

In summary, we have investigated the role of macrophage in OC expansion in a 2-AAF/PH model, employing Lip-Clod method to deplete macrophages. For the first time, we have demonstrated that depletion of macrophage results in a significant, albeit not complete, inhibition of OC proliferation after PH. In addition, we have shown that OC differentiation is attenuated after PH in macrophage depleted rats. We conclude that macrophages play an important but not crucial role in OC mediated liver regeneration in 2-AAF/PH model.

## Materials and Methods

### Animal model

Male Sprague-Dawley rats (200 g–240 g) were used. Animals had free access to standard pelleted chow and water. Rats were maintained in a temperature- and humidity-controlled room with a 12-hour light-dark illumination cycle. All rats received daily oral gavage of 2-AAF (Sigma Aldrich, St. Louis, Missouri, USA) at a dosage of 15 mg/kg for 4 days before and up to 5 days after PH. The 2-AAF was dissolved in polyethylene glycol (mol. wt. 400, Sigma). All rats were anesthetized with ether, and two-thirds PH was performed by surgical removal of the left and median liver lobes after placing a suture around the pedicles of these lobes. No gavage was performed on the day of surgery. The night before surgery, rats were deprived of food but still allowed free access to water. This study was performed in strict accordance with the recommendations in the Guide for the Care and Use of Laboratory Animals of the National Institutes of Health. The protocol was approved by the Committee on the Ethics of Animal Experiments of the Tongji Medical College. (Permit Number: 2009-S212). Every effort was made to minimize suffering.

### Liver macrophage depletion and experimental groups

Clodronate was kindly provided as a gift from Roche Diagnostics GmbH (Mannheim, Germany). Lip-Clod was prepared as previously described [28]. Macrophage depletion was performed by the so-called “suicide” technique [27]. Lip-Clod (0.1 ml/10 g) was administered during a light ether-anesthesia by injection into the tail vein 48 hours before PH. Control animals received the same volume of 0.9% NaCl intravenously under the same conditions. Rats (n = 4–5) were killed in each group at 0, 0.5, 6, 24 hours and 4, 6, 9 days after PH. Body weights were recorded daily, so were liver weights at the time of sacrifice. Restoration of liver weight was expressed as percentage of regenerated liver weight to body weight. A portion of excised liver was fixed in neutral formalin, and paraffin embedded liver tissue sections (5-μm) were used for immunohistochemical and double immunofluorescent analysis. A separate portion of liver tissue was frozen in optimal cutting temperature medium for frozen sections, and the remainder was snap-frozen and stored at −80°C for subsequent RNA isolation and protein analyses. Blood serum was collected for analysis of ALT and AST activity, using a commercial kit (Sigma).

### Cell lines and MTT assay

Liver cell line, L02, was obtained from the Cell Bank of the Chinese Academy of Sciences (Shanghai, People's Republic of China). LE/6, the oval cell line, was a kind gift from Professor Nelson Fausto. Cells were seeded in 96-well plates (6000 cells/well), and allowed to adhere for 8 hours. Lip-Clod (25 μl/ml, 50 μl/ml and 100 μl/ml) or PBS was then added to the culture medium. After incubation for 24 hours, a MTT assay was performed. The absorbance of the dissolved formazan grains within the cells was measured at 490 nm in a microplate reader (Bio-Tek Elx 800).

### Immunohistochemistry

To confirm the effect of Lip-Clod on the elimination of the macrophage population, frozen liver sections collected from each time point were stained with the monoclonal antibodies ED1 (1∶75; Serotec) and ED2 (1∶75; Serotec). The ED2 antibody recognizes mature macrophages (KCs), and the ED1 antibody recognizes both mature and immature macrophage populations. Five-μm thick frozen sections of liver were air-dried, fixed with acetone in −20°C for 10 min, and then air dried again. Endogenous peroxidase activity was blocked using 3% H_2_O_2_ in methanol for 15 min at room temperature. The sections were incubated with primary antibodies overnight at 4°C and subsequently incubated with goat anti-mouse/rabbit EnVision (Dako) for 30 min at room temperature. Both incubation steps were followed by a wash in three changes of 0.01 M PBS (pH 7.2) for 5 minutes. All reaction products were developed with diaminobenzidine and counterstained with hematoxylin.

Five-μm paraffin sections were probed for EPCAM; (1∶250, Abcam), PCK; (1∶100, Abcam) and caspase-3 (1∶200; Cell Signaling Technology). Sections were cleared with xylene, passed through a graded series of alcohols and then distilled water. Antigen retrieval was accomplished by microwaving (750 watts) slides in antigen retrieval buffer (0.01 M citric acid, pH 6.0) for a total of 20 minutes, and then cooling at room temperature for at least 30 minutes. Subsequent staining procedures were the same as described above.

### Double immunofluorescent analysis

To assess the percentage of proliferative OCs, double-labeled immunofluorescence was used to detect EPCAM and PCNA simultaneously. After deparaffinization and hydration, liver sections were microwaved for 20 minutes in 0.01 M citric acid for antigen retrieval. A cocktail of two primary antibodies was incubated overnight at 4°C, followed by incubation at 37°C for 60 minutes with fluorescein isothiocyanate-conjugated goat anti-mouse IgG and Cy3-conjugated goat anti-rabbit IgG. Nuclei were stained by 4′,6-diamidino-2-phenylindole (Sigma). All sections were analyzed using confocal laser-scanning microscopy on a Nikon Digital ECLIPSE C1 system (Nikon Corporation, Japan).

### TUNEL Analysis

To detect apoptotic cells a TUNEL assay was performed, as per the manufacturer's instructions, on paraffin-embedded liver sections using the In Situ Cell Death Detection Kit (Roche).

### Cell Scores

Periportal ductular reaction areas were measured on five randomly selected fields (×40) from HE stained sections. Areas were calculated by tracing the periportal basophilic area borders using the Image-Pro Plus 6.0 software package. These areas represented OC clusters. ED1 or ED2 positive cells were counted from 10 adjacent fields (0.4×0.32 mm^2^) at 200× magnification. OCs positive for EPCAM or PCK were counted from 20 adjacent fields (0.2×0.16 mm^2^) at 400× magnification. Apoptotic cells stained for TUNEL or caspase-3 were counted from 50 adjacent fields (0.2×0.16 mm^2^) at 400× magnification. Proliferating OCs were determined by counting cells at 200× magnification in five randomly selected periportal areas, and expressed as percentage of double-positive cells for EPCAM and PCNA to cells positive for EPCAM alone.

### RNA isolation and real-time quantitative polymerase chain reaction

Total RNA was isolated from 50 mg rat liver using TRIzol reagent (Invitrogen) according to the manufacturer's instructions. One microgram of total RNA was reverse-transcribed into cDNA template using ReverTra Ace-*α*-kit (Toyobo) according to the manufacturer's instructions. The quantitative real-time PCR was performed on cDNA samples to detect mRNA levels. The SYBR Green Realtime PCR Master Mix-PLUS (Toyobo) was used for all quantitative PCR. PCR was run on the ABI-Prism 7000 Sequence Detector system, with ABI Prism 7000 SDS Software 1.0 in 96-well format and 25 μL reaction volume per well. Primer sequences were: albumin, F: 5′GAGACTGCCCTGTGTGGAAGA3′, R: 5′ CTTTCCACCAAGGACCCACTA3′; *β*-actin, F: 5′TCCTCCTGAGCGCAAGTACTCT3′, R: 5′GCTCAGTAACAGTCCGCCTAGAA3′. Melting curves validated the utility and specificity of each primer set. Data were evaluated using the Comparative Ct Method (2^(−ΔΔCt)^) of relative quantification normalized to *β*-actin.

### Protein Extraction and Cytokine Immunoassay

Liver tissues were homogenized in extraction buffer [Tris 50 mM (pH 7.2), NaCl 150 mM, Triton-X100 containing a protease inhibitor cocktail (Complete Mini; Roche)]. The homogenate was shaken on ice for 90 minutes and centrifuged at 3000 g and 4°C for 15 minutes. Levels of TNF-*α* (eBioscience) and IL-6 (Biosource) in the supernatants were detected using a commercial ELISA kit after following the manufacturer's instructions. Protein concentration was determined using a BCA kit (Pierce), and the calculated ELISA results were normalized to protein concentration.

### Western blotting

Frozen liver samples were homogenized on ice in RIPA buffer containing protease inhibitor cocktail (Roche). Equal amounts of protein (80 μg) were separated on 10% SDS-polyacrylamide gels and transferred to PVDF membranes. Nonspecific binding was blocked with Tris-buffer saline containing 5% nonfat dry milk or bovine serum albumin by incubating the membranes for 1 hour at 37°C. The blots were then probed with antibodies against STAT3 (1∶1000; Cell Signaling Technology), phosphor-STAT3 (1∶1000; Tyr705, Cell Signaling Technology), and *β*-actin (1∶500; Santa Cruz Biotechnology) in Tris-buffered saline with 0.1% Tween 20. Membranes were washed three times and incubated with horseradish peroxidase-conjugated secondary antibodies (Pierce). Immunoreactive bands were detected with enhanced chemiluminescence (Pierce).

### Statistical analysis

Data are expressed as mean values ± standard deviation of the mean. Statistically significant differences between groups were determined by ANOVA plus post hoc Student *t*-test or Mann–Whitney U test as appropriate. *P* values of less than 0.05 were considered statistically significant.
